# Prospective of Pancreatic Cancer Diagnosis Using Cardiac Sensing

**DOI:** 10.3390/jimaging9080149

**Published:** 2023-07-25

**Authors:** Mansunderbir Singh, Priyanka Anvekar, Bhavana Baraskar, Namratha Pallipamu, Srikanth Gadam, Akhila Sai Sree Cherukuri, Devanshi N. Damani, Kanchan Kulkarni, Shivaram P. Arunachalam

**Affiliations:** 1 Department of Radiology, Mayo Clinic, Rochester, MN 55905, USA; mbmansunderbir@gmail.com (M.S.); baraskar.bhavana@gmail.com (B.B.); namrathapal123@gmail.com (N.P.);; 2 Department of Medicine, Division of Infectious Diseases, Mayo Clinic, Rochester, MN 55905, USA; anvekar.priyanka@mayo.edu; 3 GIH Artificial Intelligence Laboratory (GAIL), Division of Gastroenterology and Hepatology, Department of Medicine, Mayo Clinic, Rochester, MN 55905, USA; 4 Microwave Engineering and Imaging Laboratory (MEIL), Division of Gastroenterology and Hepatology, Department of Medicine, Mayo Clinic, Rochester, MN 55905, USA; 5 Department of Cardiovascular Medicine, Mayo Clinic, Rochester, MN 55905, USA; damani.devanshi@mayo.edu; 6 Department of Internal Medicine, Texas Tech University Health Science Center, El Paso, TX 79995, USA; 7 Centre de Recherche Cardio-Thoracique de Bordeaux, University of Bordeaux, INSERM, U1045, 33000 Bordeaux, France; kanchan.kulkarni@ihu-liryc.fr; 8 IHU Liryc, Heart Rhythm Disease Institute, Fondation Bordeaux Université, 33600 Bordeaux, France; 9 Department of Medicine, Mayo Clinic, Rochester, MN 55905, USA

**Keywords:** pancreatic cancer, cardiomyopathy, heart failure, heart remodeling, cardiac hypertrophy, early detection, ultrasound imaging, MRI, CT, cardiac sensors

## Abstract

Pancreatic carcinoma (Ca Pancreas) is the third leading cause of cancer-related deaths in the world. The malignancies of the pancreas can be diagnosed with the help of various imaging modalities. An endoscopic ultrasound with a tissue biopsy is so far considered to be the gold standard in terms of the detection of Ca Pancreas, especially for lesions <2 mm. However, other methods, like computed tomography (CT), ultrasound, and magnetic resonance imaging (MRI), are also conventionally used. Moreover, newer techniques, like proteomics, radiomics, metabolomics, and artificial intelligence (AI), are slowly being introduced for diagnosing pancreatic cancer. Regardless, it is still a challenge to diagnose pancreatic carcinoma non-invasively at an early stage due to its delayed presentation. Similarly, this also makes it difficult to demonstrate an association between Ca Pancreas and other vital organs of the body, such as the heart. A number of studies have proven a correlation between the heart and pancreatic cancer. The tumor of the pancreas affects the heart at the physiological, as well as the molecular, level. An overexpression of the SMAD4 gene; a disruption in biomolecules, such as IGF, MAPK, and ApoE; and increased CA19-9 markers are a few of the many factors that are noted to affect cardiovascular systems with pancreatic malignancies. A comprehensive review of this correlation will aid researchers in conducting studies to help establish a definite relation between the two organs and discover ways to use it for the early detection of Ca Pancreas.

## 1. Introduction

Pancreatic cancer (Ca Pancreas) is one of the leading causes of cancer-related deaths. The GLOBOCAN, in 2018, estimated pancreatic cancer to be the 11th most common cancer in the world. It is estimated that 355,317 additional instances will occur by 2040 [1]. Pancreatic cancers arise mainly from the ductal epithelial cells of the exocrine pancreas. The activation of oncogenes or deactivation of tumor-suppressing genes leads to the evolution and progression of pancreatic cancer [2]. Moreover, the molecular pathogenesis of pancreatic cancer is influenced by the disruption of several cell-regulating pathways [2,3]. There are various modifiable and non-modifiable risk factors that potentially lead to its development. However, the mutations in the biochemical makeup account for one of the strongest non-modifiable risk factors [4]. To date, several methods have been employed to diagnose Ca Pancreas. Even though using an endoscopic ultrasound with a tissue biopsy is considered the gold standard in terms of diagnosing pancreatic carcinoma, imaging modalities, like CT scanning, magnetic resonance imaging (MRI), ultrasound, and positron emission tomography (PET-CT), are also used in clinical practice.

Although the imaging modalities in use today can aid in the diagnosis and detection of pancreatic malignancies, they suffer from the limitation of identifying the disease at an advanced stage in its course. Owing to the high mortality caused by pancreatic neoplasms, there is a need to understand the impact of the tumor on the various organ systems with the hope of early diagnosis, enabling prompt treatment and improved outcomes in these patients. The heart is a four-chambered electromechanical organ responsible for blood flow and oxygen supply to the entire body. An interruption in cardiac function can cause irreversible impairments, such as brain injury, heart failure, stroke, or even sudden cardiac death [5]. Malignancies have had a significant impact on the cardiovascular system; for example, lung cancer has been shown to disrupt the conduction system [6]. Similarly, several case studies in the past have demonstrated fluctuations in the cardiac functions of patients with carcinoma of the pancreas [7,8,9]. A significant association between the heart and Ca Pancreas could be a remarkable milestone for researchers and clinicians. Given the uncertainty to date, this review aims to evaluate various factors related to pancreatic carcinoma that can affect cardiac function and its mechanics. One of the prime reasons to link any possible association of Ca Pancreas with the heart is that it is one of the first organs to receive venous blood after it circulates the gastrointestinal system. A related reason is the availability of non-invasive methods, such as electrocardiogram/seismographic sensors/echocardiography, etc., to readily assess any changes which could only become apparent with conventional detection methods after significant disease burden. This review aims to determine if there is any correlation that could be of optimal significance for physicians in clinical practice to develop methods for screening at-risk patients and diagnosing the disease early in the course.

## 2. Pathophysiology of Pancreatic Cancer

In the pancreas, a wide range of exocrine neoplasms develop, ranging from benign to malignant. In terms of pancreatic carcinoma, many genes are somatically altered or epigenetically silenced, corresponding with their sequential progression from precursor lesions. Kirsten rat sarcoma virus (KRAS) is the most frequently (>95%) altered oncogene in Ca Pancreas. It functions as a membrane-bound GTP-operated protein that normally participates in the downstream growth signaling pathway. The most often inactivated tumor-suppressor gene in this malignancy is cyclin-D kinase 2A (CDKN2A). Another commonly mutated tumor-suppressor gene in Ca Pancreas is suppressor of mothers against decapentaplegic (SMAD4), which is a member of the TGF family of surface-bound signal transduction receptors. Tumor protein 53 (TP53), a well-known cell-cycle regulator, has also been reported to be altered in the late stages of Ca Pancreas pathogenesis. All of them work together in a well-orchestrated pathway called the RAS-RAF-MEK-ERK pathway, more commonly known as the MAPK pathway. These are downstream kinases that induce various intracellular metabolic changes in response to a variety of extracellular stimuli, including growth factors, cytokines, stress, etc. These changes eventually correspond to cellular growth, proliferation, differentiation, or apoptosis [10,11]. A growing number of less common, but nonetheless important, genetic loci in Ca Pancreas have been reported to be altered, including oncogenes (AKT2: RAC-beta serine/threonine-protein kinase 2, MYB: Myeloblastosis, and MAPK: Mitogen-activated protein kinase), tumor-suppressor genes, and DNA-repair genes (GATA6, RB: Retinoblastoma, STK: Serine/Threonine kinase 11), to name a few.

Environmental factors also have a role in the pathogenesis of Ca Pancreas, with cigarette smoking being the most significant risk factor. Other factors that have a causal relationship include chronic pancreatitis, diabetes mellitus, a high-fat diet, alcohol usage, etc. Pancreatic cancer aggregation has also been reported and a rising number of inherited genetic abnormalities are known to enhance the risk. BRCA2/1, STK-11 mutations in Peutz-Jeghers Syndrome, PRSS1, SPINK1 mutations in Hereditary pancreatitis, and MLH1/MSH2 mutations in Hereditary non-polyposis colorectal cancer all increase the likelihood of developing Ca Pancreas when compared to the general population [12,13].

## 3. Current Diagnostic Techniques

Exocrine pancreatic cancer is one of the deadliest cancers. It is responsible for 3% of all malignancies and 8% of cancer-related deaths in the United States [14,15]. Pancreatic cancer symptoms are usually non-specific, such as asthenia, stomach discomfort, nausea/vomiting, and anorexia. The appearance of these symptoms at the late stage of the disease [16] makes early illness identification critical for rapid diagnosis and treatment.

Computed tomography (CT), magnetic resonance cholangiopancreatography (MRCP), and endoscopic ultrasonography (EUS) are the current modalities available for the detection of suspected pancreatic cancer and high-risk screening. The National Comprehensive Cancer Network (NCCN) guidelines for pancreatic cancer recommend germline testing for all patients with pancreatic cancer and molecular analysis for those with metastatic disease [17].

### 3.1. Computed Tomography (CT) Scan

For the initial evaluation of suspected pancreatic cancer, intravenous contrast-enhanced CT with pancreatic protocol is chosen over magnetic resonance imaging (MRI) as the first-line imaging modality. CT sensitivity varies from 76% to 96%, with larger tumors having higher levels of sensitivity. Classic CT characteristics include hypoattenuating pancreatic mass, pancreatic duct dilatation, and upstream pancreatic atrophy. Other early features of pancreatic cancer include primary pancreatic duct dilatation, sudden changes in pancreatic duct caliber, and pancreatic parenchymal alteration. In total, 5.4% to 18.4% of cases may be iso-attenuating in terms of the CT and may require an MRI or positron emission tomography (PET/CT) scan for confirmation [16].

### 3.2. Magnetic Resonance Imaging (MRI) 

Magnetic resonance imaging (MRI) has been observed to have similar or slightly lower levels of sensitivity and accuracy than a CT scan. On non-contrast-enhanced and contrast-enhanced T1 images, pancreatic cancer appears as a hypointense mass; meanwhile, on T2-weighted images, it appears as a modestly hyperintense mass [16].

### 3.3. Positron Emission Tomography (PET/CT)

FDG (18F-fluoro-2-deoxy-D-glucose) is used as a radiotracer in positron emission tomography (PET/CT). FDG accumulates in cells with a strong glycolytic metabolism. PET/CT is not deemed an alternative to CT in the diagnosis of pancreatic cancer since its relevance is uncertain. PET is not effective for staging pancreatic cancer due to the lack of spatial resolution necessary in the locoregional assessment [16].

### 3.4. Endoscopic Ultrasound

An endoscopic ultrasound is very accurate and is the gold standard for the diagnosis of pancreatic cancer; it appears hypoechoic with ductal obstruction and local invasion. Small neuroendocrine pancreatic tumors that are not seen on MRI can be detected using an endoscopic ultrasound [16].

### 3.5. Emerging Techniques for the Diagnosis of Pancreatic Cancer

Newer techniques, like radiomics/molecular imaging and machine learning, are being used to identify malignant precursors, as well as being used for earlier disease diagnoses of pancreatic cancer using computers. A considerable number of genetic anomalies in Ca Pancreas have recently been identified as a result of advances in genomic approaches. These techniques include genetic, epigenetic, non-coding RNA (ncRNA), metabolomics (study of low-molecular-weight substances and their processes), and microbiome markers. Furthermore, liquid biopsies, circulating tumor cells (CTCs), cell-free circulating tumor DNA (ctDNA), and exosomes were discovered in body fluids and are being investigated as potential tools to detect Ca Pancreas at an early stage. All of these markers, however, are in the stages of infancy and their diagnostic efficacy is yet to be validated [18]. 

Additionally, novel approaches based on artificial intelligence (AI) are being developed to increase the diagnostic accuracy of well-established techniques, like imaging (CT/MRI), pathological tissue sample analysis, and biomarker discovery. Several studies have proved the success of AI in diagnosing pancreatic carcinoma [19,20,21,22,23]. It is important to explore these emerging techniques as many methods could offer varying levels of performance. Extensive research and validation of these novel techniques will be of paramount importance in the early detection of pancreatic malignancies.

## 4. Electrophysiology and Heart Rate Variability (HRV) in Pancreatic Cancer

It is widely known that our heart is an electromechanical pump, where the generation and variation of action potential lead to the contraction and relaxation of the atria and ventricles, resulting in the completion of one cardiac cycle. The electrical activity of the heart is manifested physically as heart rate and rhythm and is very sensitive to the changes in metabolism in our body. It is hypothesized that the deranged metabolism and catabolic state in cancer patients can result in significant changes to the electrical activity of the heart, leading to heart rate variability and arrhythmias [24]. There are several known cardiovascular biomarkers, such as troponin, that are used to assess cardiac dysfunction and prognosis in cancer patients. However, another lesser-known marker, elevated heart rate (HR), has shown an association with poor prognosis in advanced cancer patients [24].

The vagus nerve, a key component of the parasympathetic nervous system, plays an important role in the variation of heart rate and rhythm. Some studies were undertaken to explore the relationship between vagal nerve activity and cancer; it has been shown that the vagus nerve inhibits excessive sympathetic activity, inflammation, and oxidative stress, which, in turn, slows tumor progression [25]. A study conducted by Couck. et al. in 2015 explored the connection between the survival of advanced pancreatic cancer patients and heart rate variability through vagal nerve activity. The study demonstrated that increased vagal nerve activity was associated with increased heart rate variability, as seen on the electrocardiogram (ECG) [25]. This study further concluded that the higher the HRV, the longer the overall survival of pancreatic cancer patients [25]. In 2016, Anker. et.al conducted a study in which a total of 145 patients with pancreatic, colorectal, and non-small-cell lung cancer were assessed for resting heart rate and compared against healthy controls. It was concluded that the resting heart rate in the cancer patients was more than 75 bpm, which was higher than the control group and was associated with a poor prognosis [26]. Moreover, in another study conducted by Anker. et.al in 2020, pancreatic, lung, and colon cancer patients were found to have increased episodes of non-sustained ventricular tachycardia, reduced heart rate variabilities, and higher baseline heart rates when compared to the control group [24]. 

All of the above studies show that an indirect correlation exists between cancers and the electrical activity of the heart, as well as the fact that variability in heart rate can predict the prognosis of such cancers, including pancreatic cancer. However, these studies could not explain that pancreatic cancer and heart rate variability have any direct cause-and-effect relationship. Currently, an observational cohort study is being conducted by the OHSU Knight Cancer Institute in collaboration with the Oregon Health and Science University, the American Association for Cancer Research, and the National Cancer Institute (NCI) to find out the legitimacy of this association and monitor heart rate variability for the early detection of pancreatic cancer [27]. 

## 5. Role of MIC-1/GDF-15 in Pancreatic Cancer and Heart Pathologies

Macrophage inhibitory cytokine-1 (MIC-1), also known as growth differentiation factor-15 (GDF-15), is a protein that belongs to the human transforming growth factor-β superfamily [28]. It is mainly secreted by the placenta in large amounts and is also secreted by a vast variety of epithelial cells in small amounts. However, its expression has been seen to be dramatically increased in certain conditions, such as cancer, inflammation, and myocardial ischemia. Therefore, it can be considered an important cytokine that mediates inflammatory reactions and is secreted in response to cellular stressors [28]. According to Bauskin et al., in 2006, MIC-1 levels increased in all types of cancers; but, marked overexpression was seen in pancreatic, prostate, thyroid, and colonic cancers [28]. In a study conducted by Koopman et al. in 2004, a combination of MIC-1 with CA19-9 was proven to be superior, when compared to CA19-9 alone, in accurately diagnosing pancreatic and other periampullary adenocarcinomas with a sensitivity of 70% and specificity of 85%. This study also showed that patients in the early stages of cancer had the highest serum MIC-1 levels [29]. This suggests that MIC-1 can play a key role in the early detection of pancreatic cancer.

Although the primary role of MIC-1 is associated with inflammatory processes, a study conducted in 2020 by Lee et al. also demonstrated the angiogenic potential of MIC-1. In this study, it was shown that the in vivo treatment of a chick embryo and mouse abdomen with recombinant MIC-1 significantly increased the surface density of capillaries and revascularization, providing a shred of strong evidence that MIC-1 can promote angiogenesis [30]. This property of MIC-1/GDF-15 and its role in mediating inflammation can serve as an important connecting link between coronary atherosclerosis and pancreatic cancer. Oxidative stress, excessive cellular proliferation, and angiogenesis are considered unifying causal factors in both diseases [31].

The development of atherosclerosis primarily depends on high inflammatory content, which then leads to lesion formation, progression, and complications. Angiogenesis exhibits a key role in the progression of atherosclerotic plaque by allowing extravasation of the plasma components, leading to an increased risk of thromboembolic complications, such as myocardial infarction [31]. A study conducted by de Jager et al. has shown that a deficiency of MIC-1 in macrophages improves the stability of atherosclerotic plaque by impairing the migration of macrophages and promoting the deposition of collagen [31]. Thus, it can be speculated that increased amounts of MIC-1 can initiate atherosclerotic lesions through proinflammation and angiogenesis and can promote the vulnerability of existing atherosclerotic plaques. This can, in turn, increase the susceptibility to recurrent cardiac ischemic events [31]. Schuab et al. conducted a prospective multicenter study to assess the relationship between MIC-1/GDF-15 and coronary artery diseases and showed that the concentrations of MIC-1/GDF-15 were significantly higher in acute myocardial infarction patients when compared to the patients admitted with other causes [32]. Another study was conducted by Bonaca et al., which demonstrated that increased levels of MIC-1/GDF-15 in patients with previous and stabilized acute coronary syndromes lead to recurrent ischemic cardiac events and complications, such as myocardial infarction, heart failure, and even death [33]. These studies demonstrate a significant correlation between MIC-1/GDF-15 and cardiac ischemic diseases.

As discussed in the above studies, high levels of MIC-1/GDF-15 are associated with ischemic cardiac diseases. However, few studies have also elicited its connection with cardiomyopathies. In the randomized controlled clinical studies conducted by Xue et al. in 2012 and Kou et al. in 2016, it was found that levels of MIC-1/GDF-15 were significantly higher in hypertensive patients with left ventricular hypertrophy when compared with the control population [34,35]. However, another study conducted by Garcia et al. showed that MIC-1/GDF-15 levels were also elevated in nonhypertensive patients with hypertrophic cardiomyopathy and that higher levels were associated with increased severity of the disease [36]. A positive correlation was also seen between the plasma levels of MIC-1/GDF-15 and the degree of myocardial fibrosis in a study conducted by Lok et al. [37]. Although all of the above studies demonstrated a significant association between MIC-1/GDF-15 and cardiac hypertrophy, the exact mechanism behind cardiac remodeling by MIC-1/GDF-15 still remains unexplained.

In conclusion, all of the above studies prove that elevated plasma levels of MIC-1/GDF-15 are significantly associated with cardiovascular diseases, cardiomyopathies, and pancreatic cancer.

## 6. IGF in Pancreatic Cancer and the Heart

Focusing on various risk factors for pancreatic cancer (smoking, obesity, lack of exercise, heavy alcohol use, family history, DM, etc.), a common culprit, i.e., insulin resistance and hyperinsulinemia, can be postulated for its development. It needs further exploration due to the frequent presence of type 2 diabetes mellitus before the diagnosis of pancreatic ductal adenocarcinoma (PDAC) [38]. This can be supported by a meta-analysis of pancreatic cancer patients, which found a relative risk of developing pancreatic cancer in patients with preceding diabetes mellitus at 1.82 (95% CI, 1.66–1.89) [39]. This analysis also found that as the duration of the diabetes mellitus increased (>5 years v/s≤ 5 years), the relative risk of developing pancreatic cancer decreased (1.5 v/s 2.1; *p* = 0.005), implying a reverse causality between pancreatic cancer and diabetes mellitus. However, it is highly unlikely that cancer with a very low (1-year) survival rate would induce diabetes years before its diagnosis [39]. The association between insulin and PDAC is a mutually reinforcing one. Ca Pancreas cells have been shown to cause peripheral insulin resistance and resulting hyperinsulinemia via the secretion of exosomes. Insulin promotes and sustains pancreatic cancer development by inducing tumorigenic inflammation, regulating lipid and glucose metabolic reprogramming, overcoming apoptosis via crosstalk with IGF-1, stimulating cancer metastasis, and activating tumor microenvironment formation (inflammation, fibrosis, and angiogenesis) [40].

Physiologically, one of the key effects of hyperinsulinemia is that it increases the bioavailability of insulin-like growth factors (IGFs). IGF-1 and insulin-like growth factor 1 receptor (IGF-1R) have been reported to be abundantly expressed in pancreatic ductal adenocarcinoma tissue. This activated insulin/IGF signaling in pancreatic ductal adenocarcinoma cells has been shown to regulate the cancer cells’ basal growth rate [41]. This pathway has been subjected to several therapeutic trials utilizing IGF-blocking drugs to stop the progression of pancreatic cancer; but, there has been little success, which may be attributable to the complex nature of this signaling system. Insulin-like growth factors (IGFs) are detected in the blood in protein-bound states with insulin-like growth factor binding proteins (IGFBPs). Patients with pancreatic ductal adenocarcinoma were shown to have excessive levels of IGFBP-1, 3, IGF2BP-2, IGF-1, and IGF1R in their blood and pancreatic cells [42,43]. Further research on these findings suggests that an increased risk of pancreatic ductal adenocarcinoma may be linked to high IGF-1/low IGFBP-3 serum concentrations. Patients with advanced pancreatic ductal adenocarcinomas and high IGF-1R to low IGFBP-3 ratio expression in the pancreas had worse overall survival rates [44]. Recent research has demonstrated that IGF and its serum-binding proteins (IGFBPs) can be used to diagnose pancreatic cancer with high specificity [45].

IGF, as a growth regulator molecule, is also related to the physiological remodeling of the heart (for example, in athletes) [46] and has a cardioprotective role in that it aids in the conversion of fibroblasts to functional cardiomyocytes after myocardial injury [47]. However, it is not unusual for the same pathways to be activated pathologically in certain circumstances, such as GH-secreting tumors, hyperinsulinemia, hypertension, and so on [48]. As insulin-like growth factor-1 (IGF-1) is a peptide with pro-hypertrophic and anti-apoptotic activities, techniques can be employed to objectively quantify any possible modifications IGF can have on cardiac musculature [48].

Surprisingly, angiotensin II, which mediates structural changes in the heart and is a component of the local renin-angiotensin system in pancreatic cancer cells, also increases IGF-IIR expression via the activation of downstream kinases, which leads to sirtuin 1 (SIRT1) degradation via the proteasomes and prevents it from deacetylating heat shock transcription factor 1 (HSF1). HSF1’s capacity to bind to the IGF-IIR promoter region was compromised as a result of increased acetylation; its increased expression induced ventricular hypertrophy and apoptosis and eventually impacted cardiac contractility [49].

## 7. ADAMTS in Pancreatic Cancer and the Heart

Pancreatic cancer is one of the deadliest cancers, with the majority of patients identified at an advanced stage when the tumor has migrated to distant places. Invasion and metastasis, like any other malignant growth, are the result of complicated interactions between cancer cells and the surrounding normal stroma. A carcinoma must first breach the basement membrane; then, it must move through interstitial tissue before entering the circulation through the vascular basement membrane. Tumor cells may accomplish this by secreting proteolytic enzymes or encouraging stromal cells to elaborate proteases [50].

A class of these proteases called disintegrin and metalloprotease with thrombospondin motifs (ADAMTS) has also been discovered in Ca Pancreas [51]. A recent study by Klç M et al. found that when compared to the normal pancreas, ADAMTS1, ADAMTS8, ADAMTS9, and ADAMTS18 were significantly detected in all malignant pancreatic samples. Furthermore, ADAMTS1, ADAMTS9, and ADAMTS18 were shown to have higher immune positivity in adjacent, as well as distant, metastatic lymph nodes as compared to normal lymph tissue. Tumor size was likewise linked to ADAMTS9 and ADAMTS18 expression in the diseased pancreas [52], implying that these proteins play important roles in the carcinogenesis and lymphatic spread of pancreatic adenocarcinoma. Similarly, ADAMTS12 is known to be involved in the progression of various cancers, such as colorectal, lung, etc., and recently, a study conducted by Song C et al. found it to be substantially overexpressed in pancreatic adenocarcinoma. A high level of ADAMTS12 expression was associated with poorer survival rates in patients with pancreatic adenocarcinoma, as well as high levels of tumor-associated macrophages, cancer-associated fibroblasts, immune checkpoint proteins, and immunosuppressive genes, all of which are part of the tumor microenvironment and aid in its progression [53].

The role of ADAMTS proteases is not limited to malignant diseases; they play important roles in a variety of benign pathologies by performing a lot of functions and participating in a variety of cardiovascular processes, such as vascular smooth muscle cell proliferation and migration, angiogenesis, vascular cell apoptosis, cell survival, tissue repair, and wound healing [54]. Omura J et al. elucidated the clinical translation of these functions in their study, which showed an upregulation of ADAMTS8 in pulmonary artery smooth muscle cells in pulmonary artery hypertension patients, promoting proliferation, ECM remodeling, and endothelial dysfunction in an autocrine/paracrine manner [55]. ADAMTS8 knockout mice demonstrated improved pulmonary hypertension and right ventricular dysfunction. ADAMTS1 is one of the most extensively studied proteinases of this family and has been found to be upregulated in human atherosclerotic lesions; its activity may lead to plaque instability [56]. Also, it is noteworthy that ADAMTS1 expression is increased by Angiotensin II (AngII) and other factors involved in vascular remodeling, representing its potential role in cardiac and vascular remodeling [57], causing clinical or subclinical ischemic cardiomyopathy, coronary artery disease, valvular or aortic diseases, and eventually affecting myocardial structure and function.

Other proteinases from the ADAMTS family have also been implicated in a myriad of cardiovascular pathologies, e.g., ADAMTS2 expression was predominantly elevated in failing human hearts [58]; the inhibition of re-endothelialization and formation of coronary plaques leading to ischemic CVD due to ADAMTS7 [59]; and elevated levels of ADAMTS7 in patients with LVEF ≤35% compared with those with LVEF >35% after acute myocardial infarction, which was independent of traditional cardiovascular risk factors and other biomarkers [60], just to enumerate a few. Their role in pancreatic pathologies has not yet been explored.

Even with the evidence of the presence of similar proteinases in pancreatic and cardiovascular pathologies, no study has been conducted to find out if pancreatic cancer ADAMTS expression can possibly affect the heart and serve as a potential molecule to detect pancreatic cancer at its early stage by either qualitatively or quantitatively measuring the changes in heart musculature.

## 8. RAS in Pancreatic Cancer and the Heart

The renin-angiotensin system (RAS), apart from its usual endocrine function in blood pressure homeostasis, is also present ubiquitously in every organ and is also constitutively expressed in pancreatic ductal carcinoma cells. Its presence in local tissues/organs and its role as an anti-apoptotic protein that influences cell growth and differentiation has been found in various animal models, as well as in humans [61].

Several molecular studies have confirmed this fact, as immunocytochemical techniques have found angiotensin II type 1 receptor (AT1R) and (pro)renin expression in beta cells of the islets of Langerhans, as well as in endothelial cells of the pancreatic vasculature [62]. Furthermore, according to an in-situ hybridization analysis which revealed that (pro)renin mRNA transcription may be restricted to the connective tissue surrounding blood vessels and reticular fibers within the islets [62], renin is released from adjacent sites of synthesis and then acts in a paracrine fashion at cellular sites of action [62]. Further downstream proteins of the system, i.e., angiotensinogen, AT1R, and ACE, regarding analysis with single-cell reverse transcriptase and Western Blotting, showed expressions of mRNA and proteins for AT1Rs and mRNA, for both angiotensinogen and ACE in human islets.

The role of the RAS in carcinogenesis can further be consolidated by the fact that RAS inhibitors (ACEi/ARB) have shown significant improvement in the overall survival (OS) of pancreatic cancer patients [63]. Also, when used along with gemcitabine monotherapy, patients have exhibited significantly better progression-free survival (PFS) and OS [64].

Similarly, RAS-blocking drugs are the cornerstone of heart failure treatment since they significantly affects the heart and are involved in cardiac remodeling due to their hemodynamic, as well as direct, cellular-level effects. Expressions of both AT1R and AT2R are found on the cardiomyocytes. The binding of AngII to AT1R induces downstream signaling pathways, including protein kinase C (PKC); mitogen-activated protein kinase (MAPK); NADPH oxidase (NOX); ROS signaling; G-protein-independent pathways, including the β-arrestin and JAK/STAT, as well as various tyrosine kinases; and scaffold proteins to regulate protein expression [65,66].

Furthermore, there is the presence of a local RAS in the heart, which can be activated by the binding of renin or prorenin to the (pro)renin receptor (PRR), which has downstream signaling mimicking that of AngII, leading to the triggering of the pro-fibrotic signaling cascade. A study conducted by Mahmud H et al. confirmed the upregulation of (pro)renin receptor (PRR) mRNA and protein expression in cardiomyopathic hearts, implicating their role in cardiac remodeling and heart failure [67].

## 9. Pancreatic-Cancer-Related Coagulopathy and the Heart

Cancer patients, in general, have a four-fold increased risk of thrombosis and venous thromboembolism (VTE). Thromboembolic disease is commonly seen in patients with pancreatic cancer, which is triggered by the formation of an intrinsic hypercoagulable state [68]. In 2016, Gedding et al. demonstrated in mice that tissue factor-positive tumor microvesicles (TF+, TMVs) are released from pancreatic adenocarcinoma cancer cells. This triggers the activation of platelets and the coagulation pathways by promoting the release of thrombin and fibrin [68,69,70,71,72,73,74].

Pancreatic cancer can manifest a variety of thromboembolic diseases, such as pulmonary embolism, deep venous thrombosis, disseminated intravascular coagulation, portal venous thrombosis, and arterial thromboembolism. Venous thromboembolism, which is a result of coagulation cascade activation, enhanced tumor growth, and angiogenesis, can directly lead to the formation of distal thrombi, eventually causing pulmonary emboli [68,75]. Pulmonary tumor emboli (PTE) are unusually seen in patients with cancer. PTE consists of tumor cells that cause the local activation of coagulation, further causing microthrombi in the pulmonary vessels. Both above-stated mechanisms cause the remodeling of the pulmonary vasculature, contributing to pulmonary hypertension, which is so far the leading cause of cor pulmonale and right heart failure [70,76].

## 10. ApoE in Pancreatic Cancer and the Heart

Cancer cells’ ability to avoid the host immune response is one of their cellular and molecular hallmarks. Tumor cells generate a wide range of changed antigens, which can be the result of mutant genes, overexpressed or aberrantly expressed cellular proteins, oncofetal antigens, altered cell surface glycolipids, or cell-type-specific differentiation antigens, etc. Cell-mediated immunity is primarily responsible for recognizing and neutralizing these antigens in cancer cells through Cytotoxic T-cells, NK cells, and macrophages. On the other hand, cancer cells elude immunity by preferentially outgrowing antigen-negative variants, by causing mutation in major histocompatibility (MHC) genes and antigen processing genes, by the secretion of immunosuppressive molecules, or by the expression of inhibitory cell surface proteins [77].

Pancreatic ductal adenocarcinoma, one of the most fatal and aggressive tumors, also employs strategies to circumvent these host defensive responses. Chen et al. demonstrated this when ApoE levels were discovered to be high in the patients’ blood, as well as pancreatic tissues, and levels correlated positively with tumor TNM staging, indicating its functional role in the disease [78]. Kemp et al., in their study conducted in 2021 on mice, revealed that ApoE expression was impacted by KRAS activation. They elicited increased ApoE+ cells after inducing KRAS and decreased number of ApoE+ cells after extinguishing KRAS expression [79]. Mechanistically, tumor-associated macrophages (TAMs) are the primary regulators of the local immunosuppressive environment in cancer stroma [80] and, accordingly, they were found to have increased the expression of ApoE in pancreatic cancer cells, which in turn lead to the expression of two chemokines, i.e., CXCL1 and CXCL5, by pancreatic cancer cells [78]. Both are known chemoattractants acting on CXCR2 receptors present on immature myeloid cells, transforming them into myeloid-derived suppressor cells (MDSCs), which are potent inhibitors of T-cell function and, thus, decrease their infiltration into pancreatic cancer cells and eventually increase their spread to distant sites [81].

According to a recent study, ApoE has a role in pancreatic cancer growth and development in addition to helping cancer cells evade the immune system. High levels of ApoE2-LRP8/c-Myc expression were found in tumor tissues and cell lines, which triggered a significant downstream kinase ERK1/2, tiding cancer cells over the G1/S and G2/M cell cycle transitions [82]. These properties make ApoE a good candidate as a tumor marker and a chemotherapeutic target in pancreatic cancer. However, a notable finding in a study conducted by Kemp et. al. also showed that expression of ApoE was higher in peripheral monocytes of pancreatic ductal adenocarcinoma patients compared to healthy individuals, suggesting that elevated monocyte ApoE expression represents a systemic response to the tumor [79].

This characteristic of ApoE’s ability to possibly affect other organs has not been explored yet. ApoE is probably required for the uptake of all types of lipids in the body. Although it helps in reverse cholesterol transport, its effects on cardiovascular events and diseases are debatable, with studies ranging from their negative cardiovascular effects to being neutral or having a protective role. However, certain isoforms have been shown to have detrimental effects on cardiovascular health. For example, ApoE2 increases atherogenic lipoprotein levels (it binds poorly to LDL receptors) and ApoE4 increases LDL levels (it binds preferentially to triglyceride-rich, very low-density lipoproteins, leading to the downregulation of LDL receptors) [83]. Consequently, it may be inferred that determining the genotype may be more beneficial in clinical practice than measuring plasma ApoE levels. Its elevated levels in pancreatic cancer patients warrant further investigation into whether or not it has a role to play in the development, acceleration, or reduction of cardiovascular diseases; this can serve as a potential source of cancer detection through its effects on the cardiovascular system.

## 11. SMAD4 Mutations and MAPK Cascade Involvement in Cardiac Pathology

A few studies have shown that SMAD 4 gene inactivation causes pancreatic cancer with distant metastases and a poor prognosis [84,85,86,87,88]. Furthermore, SMAD 4 inactivation mutations are known to induce cardiac fibrosis. Jian Wang et al. reported, in 2005, that targeting SMAD4 resulted in cardiac hypertrophy and shortened longevity in mice [89]. Cardiac hypertrophy was followed by ventricular remodeling due to maladaptive fibrosis, which resulted in a considerable decrease in cardiac contractility. Another potential culprit which acts on both organs, i.e., the heart and pancreas, is TGF. It is one of the upstream growth factors that uses SMAD-4-dependent intracellular signaling to induce cardiac fibrosis, as well as pancreatic stellate cell (PSC) mediated fibrosis. It is extensively secreted by PSCs in the tumor milieu and is responsible for poor prognosis and early spreading in PDAC [90,91]. This was demonstrated by a reduction in fractional shortening (FS), an echocardiographic marker of cardiac contractile function [92].

Similar to how colorectal cancer progresses from non-neoplastic epithelium through adenoma to invasive carcinoma, it is thought that invasive pancreatic tumors develop from clearly characterized non-invasive precursor lesions. Although various mutations involving oncogenes and tumor-suppressing genes in pancreatic cancer cells occur sequentially and parallel to the gross and microscopic stages, the accumulation of these mutations is more important than their occurrence in a specific order [93]. One of the earliest oncogene mutations occurring in these cells, i.e., Kirsten rat sarcoma virus (KRAS), leads to the phosphorylation and constitutive activation of downstream signaling pathways. This notably includes Phosphoinositide 3-kinase/Protein kinase B and Mitogen-activated protein kinase (PI3K/Akt and MAPK), which augment cell growth and survival [94]. MAPKs are involved in a diverse repertoire of biological events, such as proliferation, differentiation, metabolism, motility, survival, and apoptosis. These biological processes are the result of signal transduction and are controlled by four MAPK subfamilies in particular. These include extracellular signal-regulated kinases (ERK1/2), c-Jun NH2-terminal kinases (JNK1, -2 and -3), p38 kinase (α, β, γ, δ), and big MAPK (BMK or ERK5) [95]. Similarly, in response to extracellular signals, the PI3K/Akt pathway controls cell metabolism, growth, proliferation, and stress responses [96]. It is worth noting that in a study conducted by Xu et.al. in 2019, one of the novel driving factors for pancreatic cancer pathophysiology, IGFBP-2 (as discussed above), was found to be overexpressed and statistically reduced overall survival (OS) periods in the Ca Pancreas patients. It stimulated cancer cell growth by activating the PI3K/Akt signaling pathway [43]. Fu et al., in 2020, found another protein called transmembrane protein-158 (TMEM 158) to be overexpressed in pancreatic cells, which correlated with higher TNM staging, tumor size, and invasiveness. This protein also utilized the PI3K/Akt and TGFβ-1 pathways [96].

The relatively common presence and functionality of these pathways in every organ make them important targets for pancreatic cancer management. For instance, mechanical overload, neurohormonal stimulation, and oxidative stress are all prominent causes of cardiac hypertrophy. This might be a compensatory reaction to increase contractility and keep cardiac output stable without causing disease. However, when stressors continue, this compensatory mechanism can evolve into a decompensated state, resulting in dramatic alterations in gene expression profile, contractile failure, and extracellular remodeling [97]. All of the major subfamilies of MAPK mediate these processes, ranging from cardiac hypertrophy to remodeling, with the predominant effect being skewed towards the pathway/subfamily activated.

In terms of extracellular signal-regulated kinase (ERK1/2), in a study conducted by Kai H. et al. in 1998, this protein expression in hypertrophic cardiomyopathy (HCM) patients showed a positive correlation with the severity of hypertrophy. Apart from the obvious mechanical effects, it induced sarcoplasmic reticulum calcium channel abnormalities and arrhythmias by altering ion channels, exchangers, and pumps, serving as a potential contributor to contractile defects and sudden cardiac arrest [98].

JNK has been implicated in promoting cardiac remodeling downstream of various pathways; but, its role in hypertrophy is not clear [99].

P38 seems to play a vital role in heart remodeling following injury. In 2001, Liao et al. observed that targeted p38 activation in the heart causes restrictive cardiomyopathy with substantial interstitial fibrosis [100]. In a separate study conducted on cultured rat cardiomyocytes, it was found that enhanced activation of p38 MAPK had negative inotropic effects, which were mediated by decreasing the myofilament response to Ca ^(2+)^ [101].

Similarly, the PI3K/Akt pathway is crucial in the regulation of cell development and survival, e.g., in cardiomyocytes. It has been demonstrated that activating the PI3K/Akt pathway alleviates the unfavorable post-infarct alterations in the myocardium. However, this effect is blunted by diabetes mellitus [95], which is one of the factors present preceding the diagnosis of pancreatic cancer.

## 12. Role of CA 19-9

Carbohydrate antigen 19-9 (CA 19-9) has been popularly used as a biochemical marker for pancreatic cancer. However, CA19-9 has also been seen to affect the heart. An independent and positive association was established between CA 19-9 and coronary artery calcification and arterial stiffness by Park et al. in 2019 [102]. Another study by Windecker et al. in 2005 demonstrated the statistical significance between increased levels of CA 19-9 and heart failure [103]. Shi et al. found a positive correlation between CA 19-9 levels and NT-proBNP quartiles. Furthermore, they also found a significant association between increased tumor biomarkers and all-cause mortality, hospitalization due to heart failure, and cardiovascular (CV) mortality [104].

## 13. Discussion

Diagnosing Ca Pancreas at an early stage is a challenge as well as an unmet need in the current evidence-based medicine era. Although pancreatic cancer accounts for only about 3% of all cancers in the US and about 7% of all cancer deaths, with a steady rise in incidence of 0.5% to 1.0% per year and a trajectory to become the second-leading cause of cancer-related mortality by 2030, it becomes even more critical to diagnose it early in its course [105]. There are numerous difficulties in the early detection of Ca Pancreas in clinical practice since the symptoms of individuals with early-stage Ca Pancreas are uncommon and non-specific.

Presently, an observational cohort study is being conducted, as mentioned in the above sections, to monitor heart rate variability for the early detection of pancreatic cancer. Although the results are yet to be assessed, it gives an insight into the fact that the exploration of this relationship can be worthwhile and, if a definite association exists, heart variability can serve as one of the promising criteria in the early detection of pancreatic cancer, along with current biomarkers and novel metabolomic markers.

Taking a cue from the above-mentioned study and molecular level advances to detect Ca Pancreas, we searched further along its course of natural history to find potential targets that could link Ca Pancreas to the heart as it is the foremost organ that receives metabolites, toxins, or products secreted by pancreatic tissue through the inferior vena cava after it passes through the liver. This makes the heart vulnerable to any potential effects these metabolites or toxins can have on the cardiac physiology, valves, musculature, and motion.

Starting with predisposing conditions and risk factors, the that chronic inflammatory states (infectious and non-infectious etiology) and cancer have a cause-and-effect relationship was established long ago [106]. Alcohol consumption and chronic pancreatitis are two acknowledged risk factors for Ca Pancreas that mediate their effects through inflammation, lending credence to the aforementioned assertion. While assessing for an inflammatory marker, MIC-1/GDF-15 levels were found to be elevated and significantly associated with cardiovascular diseases, cardiomyopathies, and pancreatic cancer in multiple studies. Hence, it can serve as a potential diagnostic tool in the early identification of pancreatic cancers as any patient presenting with premature coronary artery disease or cardiac hypertrophy and dysfunction, and/or without any obvious risk factors, could be evaluated for MIC-1/GDF-15 plasma levels, which might lead to early detection of pancreatic adenocarcinomas.

Further exploring known Ca Pancreas mutations in proto-oncogenes, oncogenes, and tumor-suppressor genes revealed that SMAD-4 had a plausible association with cardiac fibrosis, cardiac muscle hypertrophy, and decreased contractility, which was objectively measured using echocardiography. Similarly, two downstream kinase pathways, i.e., MAPK and PI3K/Akt, were found to be associated with the pathological remodeling of the cardiac musculature.

However, one can argue about the meager chances of the simultaneous presence of these mutations in Ca Pancreas cells and heart cells as no study has been completed, to date, to explore this. But, a source of potential crosstalk exists in the form of a growth factor, i.e., IGF-1, which was found to be significantly overexpressed, along with its receptors on Ca Pancreas cells. Also, a study conducted by Hirakawa et al. in 2013 showed that increased levels of free IGF-1 and its receptor IGFR-1 (high IGF-1/low IGFBP-3 and high IGF-1R/low IGFBP-3) were associated with a more aggressive form of the disease. IGFs also use the above-mentioned downstream kinase pathways, i.e., MAPK and PI3K/Akt, for modulating their effects on the heart musculature, which spans from normal physiologic remodeling to pathologic remodeling under the influence of various stressors and molecules, including insulin. Patients with Ca Pancreas usually have hyperinsulinemia preceding the diagnosis. Thus, more research is needed to investigate the significance of IGFs produced in the context of Ca Pancreas in inducing physical/biochemical changes in cardiac musculature, as well as a method to tap it.

Ca Pancreas is usually diagnosed very late due to early metastasis and non-specific symptoms. Just like in any other carcinoma, Ca Pancreas cells mobilize themselves through the surrounding stroma via proteases and, during our review, it was noted that ADAMTS1, ADAMTS 8, ADAMTS 9, and ADAMTS 18 were highly expressed by them and associated with early nodal, as well as distant metastasis. Of these, ADAMTS1 was noted to cause plaque instability, predisposing the individuals to ischemic cardiomyopathies and infarctions. ADAMTS8 was found to affect the right ventricle through the development of pulmonary arterial hypertension, causing its hypertrophy and motion abnormalities. On the same note, the formation of pulmonary tumor emboli and a hypercoagulable state in Ca Pancreas patients can cause a plethora of thromboembolic diseases, including the formation of microthrombi in the pulmonary vessels. Both methods promote pulmonary vascular remodeling, which leads to pulmonary hypertension and, in certain cases, may cause cor pulmonale and right heart strain/failure.

Interestingly, during our review, we found that a systemic neurohumoral system (renin-angiotensin system), which plays a key role in homeostasis and heart remodeling during heart failure, operated autonomously in the Ca Pancreas milieu. (Pro)renin was found to be expressed by pancreatic cancer stromal cells and the endothelium of pancreatic tissue vasculature. Angiotensinogen, AT1R, and ACE protein expression were also identified in pancreatic islet cells. Mirroring this, the renin-angiotensin system also operates locally in the heart musculature, along with its systemic activation in heart failure patients. This local RAS could be activated by (pro)renin and it used some of the same downstream kinases as used by IGF, i.e., MAPK and PI3K/Akt, to induce cardiac remodeling and pro-fibrotic changes. Although the role of the RAS and the drugs acting on it are being researched regarding potential treatment options for pancreatic cancer, the above-mentioned evidence of the presence of similar signaling pathways in the pathophysiology of both pancreatic cancer and heart failure warrants further exploration and possible cross-talk between the two organs as a way of diagnosing pancreatic cancer in the early stages, by either detecting cardiac motion abnormalities or remodeling.

Upon further review of a characteristic feature of cancer cells being to evade the host immune system for proliferation, we found that a lipoprotein molecule (ApoE) expression on Ca Pancreas cells was being positively influenced by one of the earliest oncogene mutations (KRAS) present in the Ca Pancreas, which led to the development of a more advanced disease with higher TNM staging. Although the effects of ApoE on the heart are variable, certain isoforms, like ApoE2 and ApoE4, are known to be atherogenic. Further studies need to be conducted to find out if the altered expression of this lipoprotein in Ca Pancreas can exacerbate pre-existing coronary artery disease and acute coronary syndrome or bring about ischemic changes in the myocardium.

Lastly, the well-known biomarker of Ca Pancreas, CA19-9, was found to have an independent association with coronary artery calcification and heart failure. The effect of markedly raised CA19-9 levels in Ca Pancreas has not been studied at all with respect to cardiovascular diseases.

**Figure 1:**
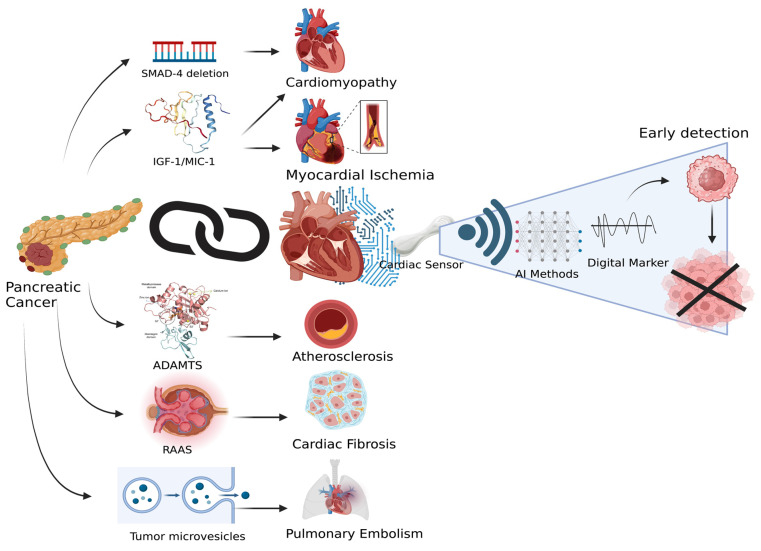
Molecular targets from pancreatic cancer affecting the heart and the use of cardiac sensors and artificial intelligence (AI) to develop a novel digital marker (created using Biorender.com, accessed on 12 June 2023).

All of the aforementioned data points toward the possibility that these molecules have an impact on the physical and maybe electrical activity of the heart, whether directly or indirectly. It is possible to contend with that because there are no known direct studies or cause-and-effect relationships and these molecules, singularly, may not have a significant impact on the heart. However, there is sufficient evidence to show that, if these molecules act in confluence, they can cause detectable changes in the heart’s musculature and contractility, as summarized in Figure 1.

These changes can have a definitive cardiac signature, which can be traced using cardiac sensors and assessed using various AI models for the early detection of pancreatic cancer. The currently available imaging modalities, such as CT, MRI, and echocardiography, can also be used to objectively evaluate these alterations. However, due to the relatively aggressive nature of cancer, there is very little time for any possible physical changes to manifest themselves enough to be clearly detected by the human eye. This issue can be addressed by using seismographic sensors that act similarly to the vestibular apparatus found in the inner ear of the human body, which senses any minute linear or rotational head movements and adjusts the body posture accordingly [107]. This technology is currently being used for fall evaluation in elderly patients, precisely delivering radiation to tumors after counterbalancing breathing and organ movement and allowing remote patient surveillance [108]. Sensors with similar functionality can be devised to detect any changes in the heart motion or musculature due to pancreatic cancer; data retrieved from them can be processed to develop a novel digital marker for the early detection of pancreatic cancer. The linear, as well as rotational, motion of the heart in the chest cavity can be mapped using accelerometers and gyroscopic sensors built into a patch. Studies can be conducted first to identify normal cardiac signature signals, followed by those of pancreatic cancer patients, to eventually form a data registry. This can be used to train various data learning models to eventually assess and diagnose Ca pancreas through cardiac sensors.

## 14. Conclusions

The currently available and emerging diagnostic techniques help in the identification of pancreatic cancer after the development of symptoms, at the advanced stages when the disease has already reached an incurable stage. In this article, we aimed to identify the molecular modalities associated with pancreatic cancer that affect the heart and manifest physically as changes in cardiac musculature through hypertrophy/fibrosis/remodeling, eventually causing cardiac motion abnormalities. Among the reviewed factors, MIC-1, SMAD4, and IGF-1 supported a possible association between Ca Pancreas and cardiac abnormalities; they have potential worth exploring. Although indirect, different subtypes of ADAMTS and coagulation cascade also showed a correlation. Future studies should be conducted to explore the cause-and-effect relationship between pancreatic cancer and the heart as this might prove to be a milestone in the early detection of pancreatic cancer.

## Data Availability

The review was based on publicly available academic literature databases.
